# Mr. William H. S. Wood

**Published:** 1908-01

**Authors:** 


Mr. William H. S. Wood, of New York, senior member of the firm of William Wood & Company, died at his home, December 11, 1907, aged 67 years. He was the son of William Wood and the grandson of Samuel S. Wood, the latter being the founder of the great publishing house of Samuel S. and William Wood, which later assumed the name of William Wood & Company. In 1866, he established the *Medical Record,* Dr. George F. Shrady becoming its first editor. By a singular turn of fate these two distinguished men passed away within a few days of the same time. Later, Mr. Wood purchased the *American Journal of Obstetrics,* then edited by Dr. Paul F. Mundé and now by Dr. Brooks H. Wells. These two medical periodicals under these editors and publishers each soon became the greatest of their class and have so remained until the present time. Mr. Wood was an active citizen taking interest in public affairs and becoming a member of many important societies. He was noted for his philanthropic liberality as well as for his sterling integrity of character and tender heartedness toward those who needed aid and counsel. A wife, three sons and a daughter survive.

